# Two cases of pancreatic arteriovenous malformations treated with coil embolization via percutaneous transhepatic portal vein approach

**DOI:** 10.1016/j.radcr.2025.09.038

**Published:** 2025-10-08

**Authors:** Yu Sasaki, Hiroki Okada, Hiroto Imaimatsu, Kodai Hujihara, Kai Ishigaki, Takuji Araki

**Affiliations:** Department of Radiology, University of Yamanashi, 1110 Shimokato Chuo, Yamanashi, Japan

**Keywords:** Coil embolization, Pancreatic arteriovenous malformation, Percutaneous transhepatic portal vein approach, Case report

## Abstract

Endovascular treatment for pancreatic arteriovenous malformation (P-AVM) has primarily involved transarterial approach. Here, we present two patients with P-AVMs, both of whom were treated using a percutaneous transhepatic portal vein approach in which coil embolization of the aneurysmal draining vein was conducted without arterial embolization. Contrast-enhanced computed tomography revealed a P-AVM with several feeding arteries and minimal veins, with Yakes types IIIa and IIIb presented in Cases 1 and 2, respectively. In Case 1, no residual AVM was observed. In Case 2, several residual feeding arteries were observed immediately post-embolization; however, they further reduced in number after 4 months. In Case 2, the portal venous pressure decreased from 22 mmHg to 14 mmHg. No adverse events were observed during the follow-up.

## Introduction

Pancreatic arteriovenous malformation (P-AVM), first reported by Halpern et al. in 1968 [1], is a relatively rare disease. P-AVMs are mainly congenital and often associated with Osler-Weber-Rendu Syndrome (hereditary hemorrhagic telangiectasia); however, P-AVMs developing secondary to trauma or surgical procedures have been reported. P-AVMs can cause severe complications, such as gastrointestinal bleeding, acute pancreatitis, and refractory portal hypertension [2], and when a P-AVM enlarges, treatment is more challenging and invasive. Reports concerning P-AVMs treated via percutaneous arterial embolization rather than via surgical operations have recently increased owing to the development of various devices and imaging modalities [3,4]. Endovascular treatment has been primarily via embolization of the pancreatic arteries instead of the portal vein. However, complex vascular anatomy and adverse events, including pancreatic ischemia, sometimes make arterial embolization highly challenging. Herein, we report two cases of P-AVMs successfully treated with coil embolization via the portal vein without arterial embolization using a percutaneous transhepatic portal vein approach.

## Case presentation

### Case 1

**Fig. 1:**
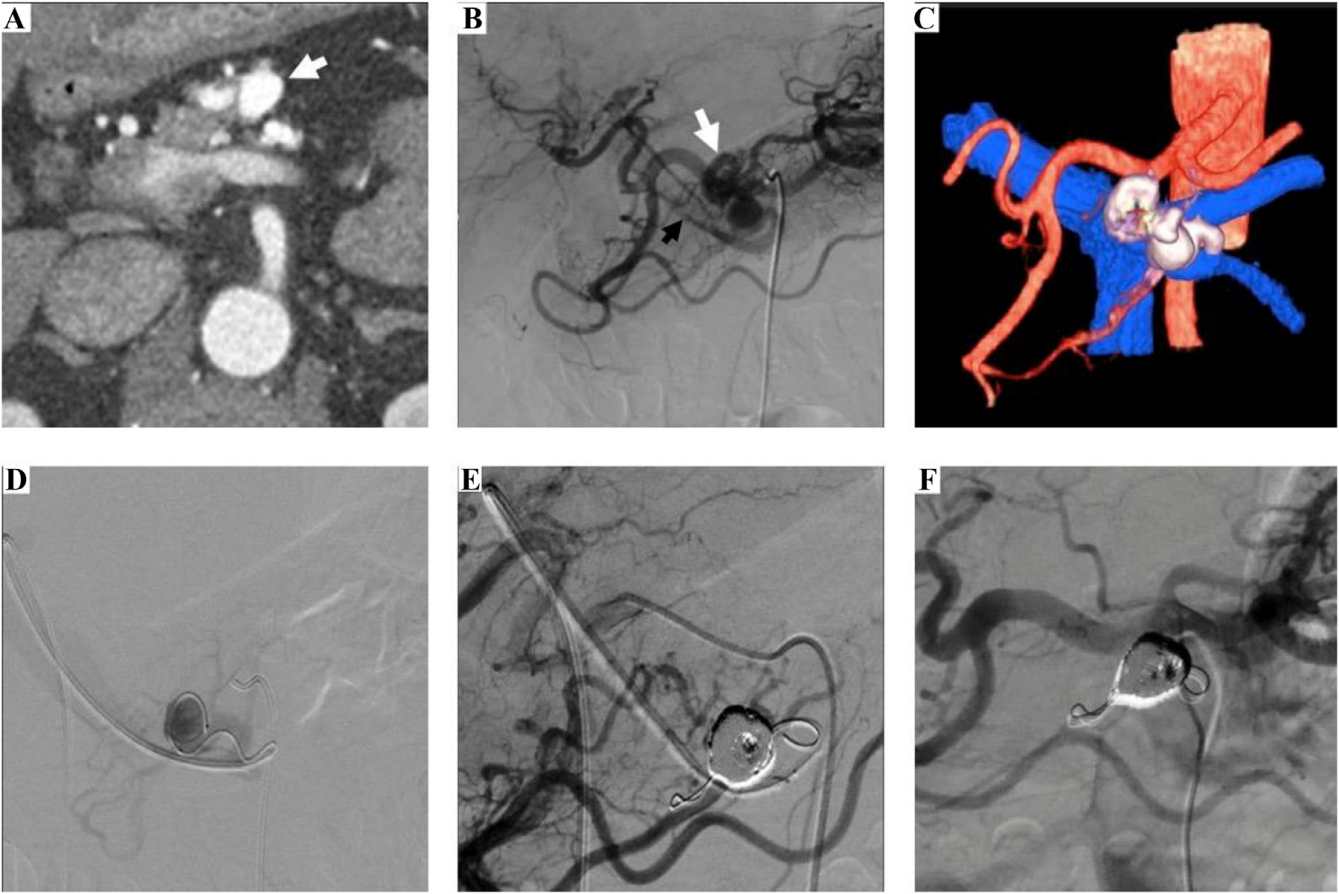
A 54-year-old male. (A) CT tomography revealing abnormal vessels in the pancreatic body (white arrow). (B) Celiac arteriography revealing type IIIa P-AVM with multiple feeding arteries draining into the aneurysmal vein (white arrow) that connects to the portal vein (black arrow). (C) 3D rendering of the CT images. (D) Venography of aneurysmal sac revealing a single main draining vein and a few microscopic collaterals. (E) Common hepatic arteriography after embolization revealing that the feeding arteries decreased slightly but remained, with no residual aneurysmal vein or early portal venous appearance. (F) Celiac arteriography revealed further decrease in the feeding arteries after four months.

A 54-year-old male, who initially presented with difficulty speaking, had a very small infarct in the right thalamus. He was managed conservatively and discharged. However, his impaired glucose tolerance, which was identified at that time, did not improve with dietary guidance or medical management. Therefore, contrast-enhanced computed tomography (CT) was performed to investigate the presence of any structural abnormalities. The CT findings indicated abnormal vascular development in the pancreatic body. He had no family history of vascular diseases, surgery, or abdominal trauma. On admission, laboratory data were unremarkable, except for an elevated HbA1c level. Arterial-phase contrast-enhanced CT revealed abundant small blood vessel hyperplasia in the pancreatic body (Fig. 1A) without a solid mass. Celiac arteriography showed abnormal small-vessel growth, similar to the CT findings. Feeding arteries from the dorsal pancreatic and anterior and posterior superior pancreaticoduodenal arteries connected the aneurysmal draining vessel to the portal vein (Fig. 1B). Multiple feeding arterioles and a single aneurysmal, winding draining vein were observed (Fig. 1C). This abnormal condition was diagnosed as Yakes type Ⅲa Angiography showed multiple feeding arteries, and arterial embolization was considered to be challenging. Therefore, aneurysmal venous sac embolization from the portal vein via percutaneous transhepatic approach was considered a suitable treatment option.

Percutaneous portal access from the umbilical portion was achieved using ultrasonography to insert a 5 French (Fr) sheath (Super sheath 5Fr 25 cm, Medikit, Tokyo, Japan). The aneurysmal venous sac was selectively catheterized from the splenic vein branch using a balloon catheter (Selecon MP Catheter II 5.2Fr, Terumo, Tokyo, Japan) and a microcatheter (Progreat β3 2.2Fr, Terumo, Tokyo, Japan). Venography of the aneurysmal sac revealed a single main draining vein and a few microscopic venous collaterals (Fig. 1D). Sac-packing embolization was conducted using several detachable microcoils (Target 360; Stryker). The puncture tract of the liver was embolized using elongated gelatin particles. On celiac arteriography immediately post-embolization, a considerable decrease in the number of feeding arteries was observed, with no residual aneurysmal sac, and no early portal venous appearance (Fig. 1E).

At 4 months postoperatively, celiac arteriography showed complete disappearance of the residual P-AVM and the feeding arteries (Fig. 1F). Prior to embolization, portal venous pressure was 7 mmHg. Post-embolization, the wedged hepatic venous pressure was 7 mmHg. No adverse or clinical events were observed during the follow-up period.

### Case 2

A 59-year-old female was diagnosed with impaired glucose tolerance during a health check-up. At the patient’s request, a contrast-enhanced CT scan was performed at her own expense to screen for any structural lesions. This was her first health check-up, and she had no subjective symptoms. Contrast-enhanced CT revealed abnormal vascular development in the pancreas from head to tail.

**Fig. 2:**
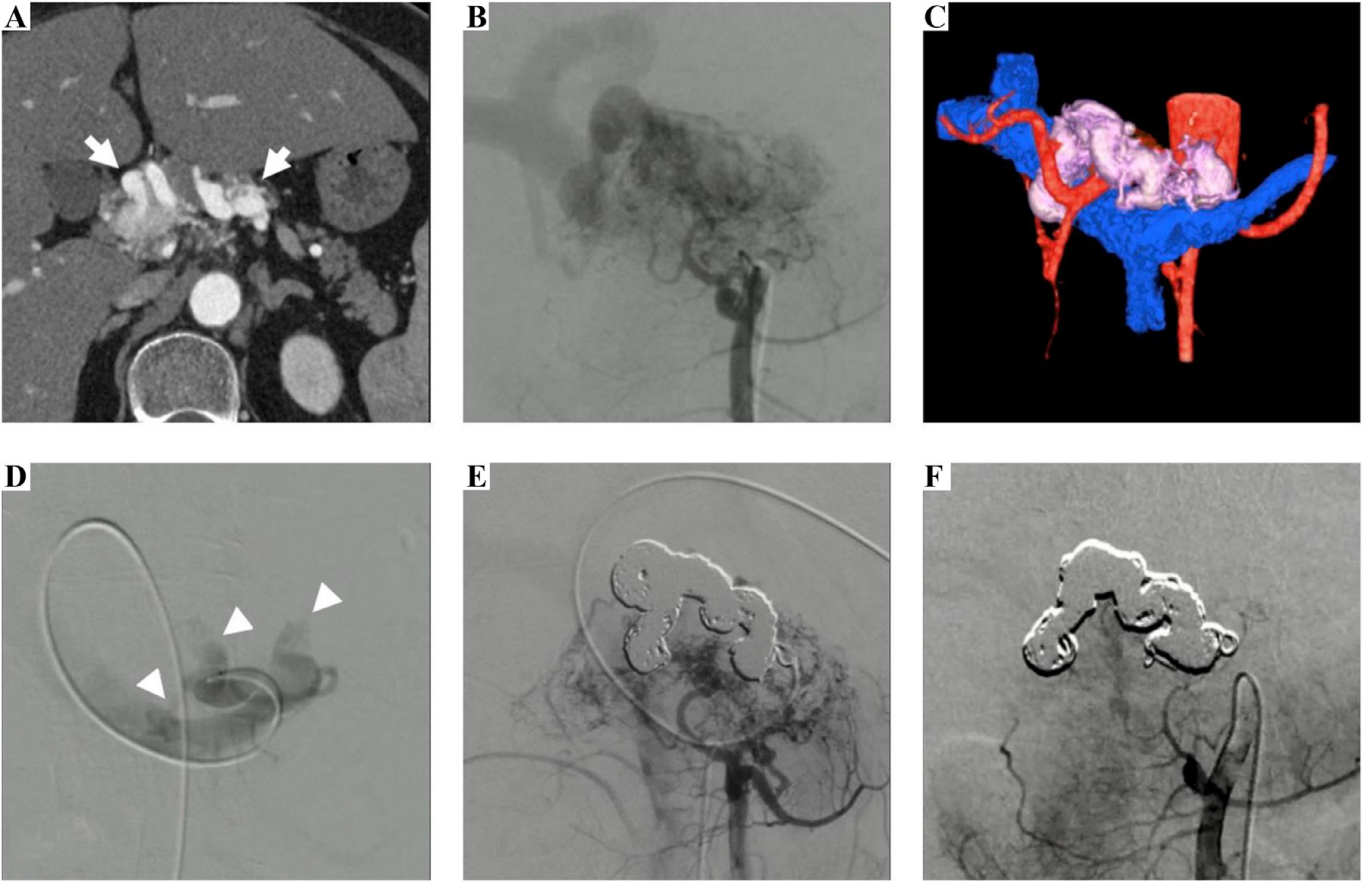
A 54-year-old female. (A) CT tomography revealing abnormal pancreatic vessels (white arrows). (B) Superior mesenteric arteriography revealing a type IIIB pancreatic AVM with multiple feeding arteries draining into the aneurysmal vein trunk that drains into the portal vein. (C) 3D rendering of the CT images. (D) Portal venous access and selective angiography of the aneurysmal vein trunk revealing the three main draining veins (white arrowhead). (E) Regional packing coil embolization of the entire aneurysmal vein trunk. Superior mesenteric arteriography after embolization revealing no residual aneurysmal vein trunk, a portion of residual P-AVM, and a decrease in the early portal venous appearance. (F) Superior mesenteric arteriography revealing further decrease in the feeding arteries and nidus after four months.

She had no relevant family history or history of trauma. Laboratory data were unremarkable, except for an elevated HbA1c level. Arterial-phase contrast-enhanced CT revealed blood vessel hyperplasia from the pancreatic head to the tail, with a winding, aneurysmal draining vein trunk (Fig. 2A).

Celiac and superior mesenteric angiography revealed several feeding arteries, including the posterior-superior and anterosuperior, inferior pancreaticoduodenal, dorsal pancreatic, and proper and common hepatic arteries converging to an aneurysmal vein trunk, wherein blood flowed out through branch veins into the splenic and left gastric veins. This condition was diagnosed as Yakes type IIIB P-AVM (Fig. 2B, C).

**Fig. 3:**
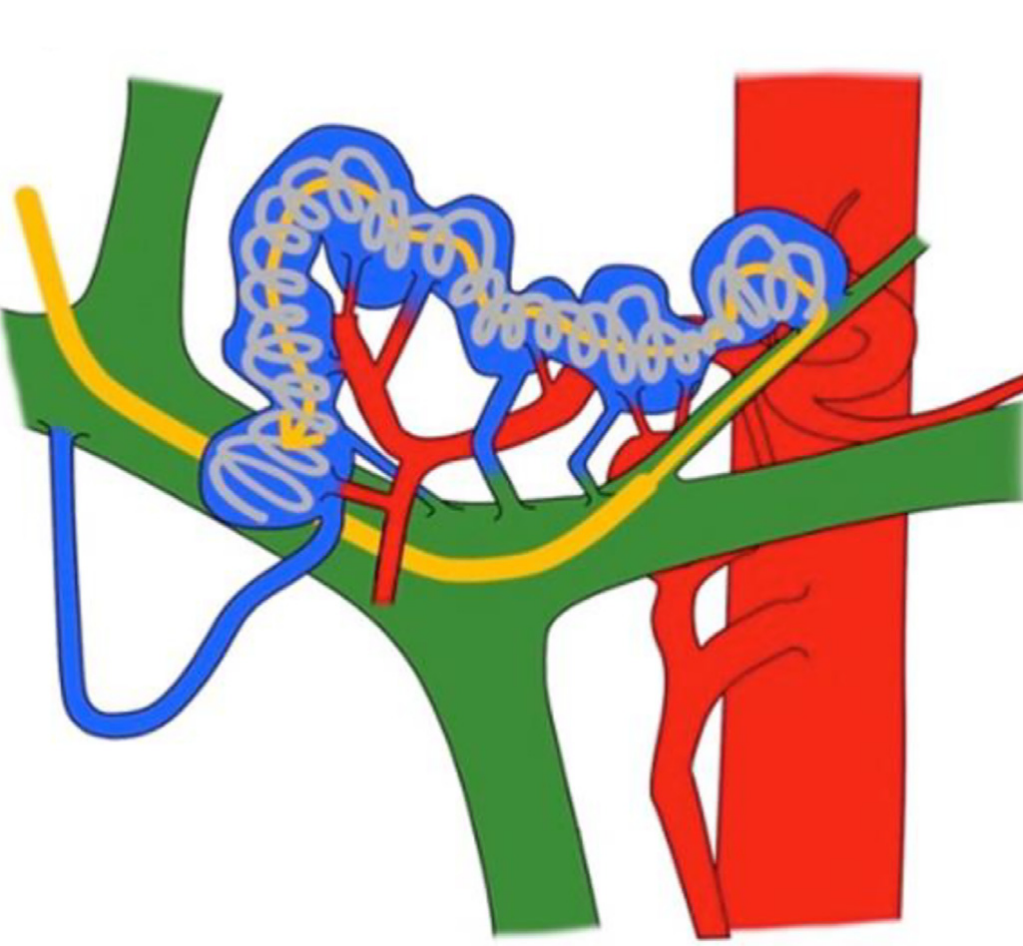
The illustration of packing coil embolization on whole aneurysmal vein trunk in Case 2.

A percutaneous portal access was achieved from the umbilical portion under ultrasound using a 5 Fr sheath (Super sheath^Ⓡ^ 5Fr 25 cm, Medikitt, Tokyo, Japan). The aneurysmal vein trunk was selectively catheterized from the splenic vein branch, using a 4Fr C2 catheter (Medikitt, Tokyo, Japan), to insert a microcatheter (Carnelian HF^Ⓡ^ 2.7, Tokai Medical Products, Aichi, Japan) to the end of the dilated draining vein. Long regional packing coil embolization of the long aneurysmal vein trunk was conducted to cover a number of flowing-out veins through several detachable microcoils (Ruby coil STANDARD, POD Packing coil, POD, Medico’s Hirata, Tokyo, Japan. Target XL 360 soft. Target Standard) (Fig. 2D, Fig. 2, Fig. 3).

On celiac and superior mesenteric arteriography, which was performed immediately post-embolization, no residual aneurysmal vein trunks, decrease in feeding arteries, and early portal venous appearance was observed. A few residual feeding arteries remained, including the dorsal pancreatic artery and a portion of the P-AVM (Fig. 2E). Prior to embolization, portal venous pressure was 22 mmHg. Post-embolization, the wedged hepatic venous pressure decreased to 14 mmHg.

At 4 months postoperatively, celiac and superior mesenteric arteriography findings indicated that only a few residual feeder arteries remained and that the aneurysmal vein trunk had disappeared (Fig. 2F). No adverse clinical events occurred during the follow-up period.

## Discussion

As first described by Halpern in 1968, P-AVM is an abnormal vessel anastomosis between the arterial and venous systems within the pancreas [1]. Congenital causes of P-AVM are associated with remnants of primary vascular network or Osler's disease, while acquired causes are associated with portal hyperplasia owing to cirrhosis, pancreatitis, neoplastic disease, and traumatic injury. P-AVMs can cause complications, such as chronic pancreatitis, glucose intolerance, acute pancreatitis, duodenal ulcers, bleeding, portal hypertension owing to the blood steal phenomenon, blood pressure differences, and rupture [2,5]. Total or partial pancreatectomy is the optimal treatment for a complete cure; however, there is a risk of severe complications, such as massive intraoperative bleeding, hypoglycemia, and pancreatic juice leakage [7,8].

**Fig. 4:**
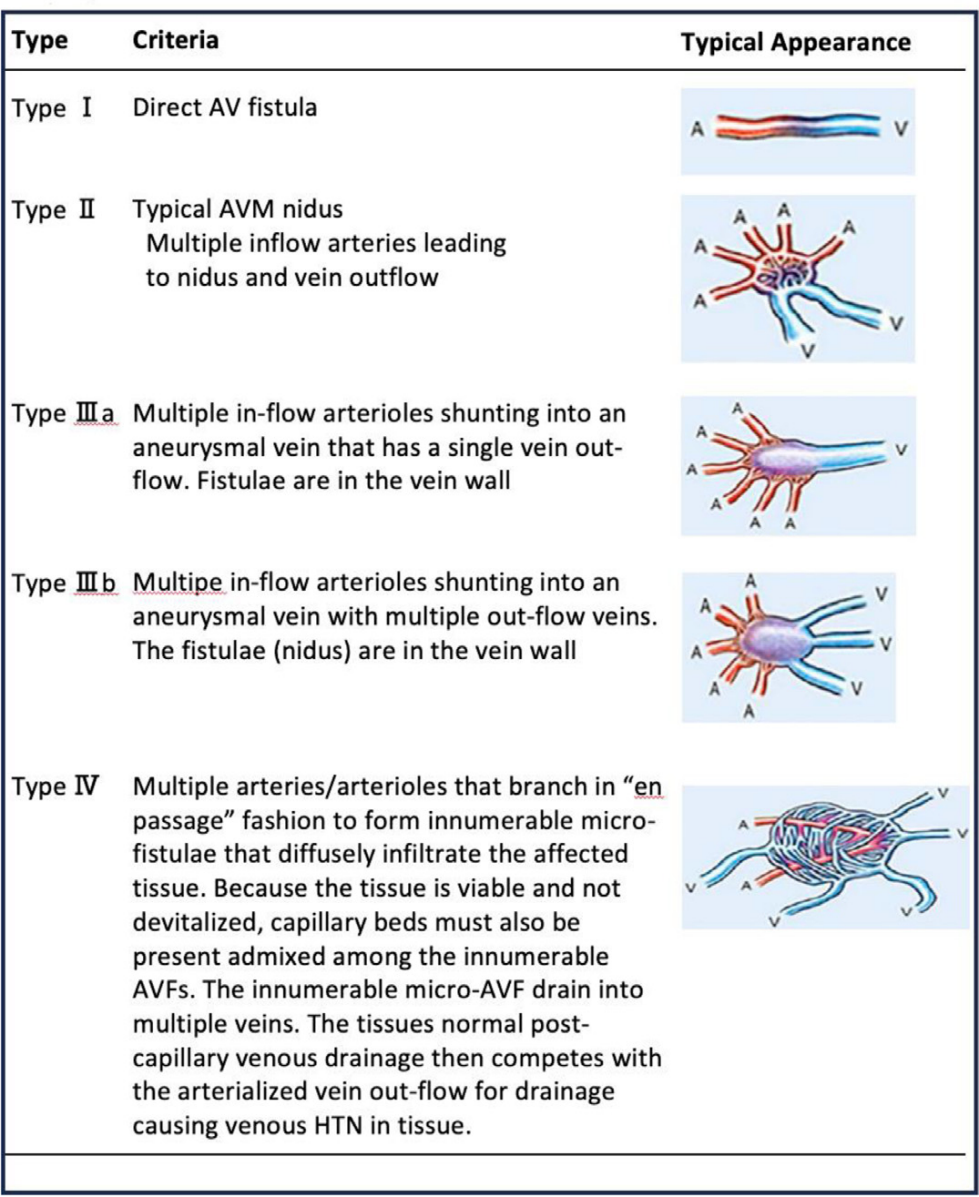
Yakes AVM classification [10].

Recently, the number of reports concerning P-AVMs treated with percutaneous embolization has increased. Most percutaneous treatments for P-AVMs involve transarterial embolization with coils or N-butyl cyanoacrylate (NBCA) glue embolization. However, for large feeding arteries, residual issues and adverse events, such as gastric ulcers, acute pancreatitis, portal vein thrombosis, and bowel ischemia have been reported [9]. Conversely, the transvenous approach tends to be selected when transarterial approaches have failed. However, in some cases corresponding to Yakes type Ⅲa or Ⅲb, complete treatment can be achieved using only a transvenous approach [6]. In venous embolization for AVMs of Yakes type Ⅲa or Ⅲb, it is crucial for curative treatment that all draining venous trunks with multiple outflow veins are completely occluded with embolic materials. NBCA glue embolization may be used in addition to coils; however, liquid embolic materials were not utilized in these two cases because of concerns relating to pancreatic ischemic complications. Accurate diagnosis of P-AVMs is essential for selecting the optimal therapeutic approach and predicting treatment outcomes. The Yakes classification is useful for determining the appropriate treatment strategy [10] (Fig. 4).

There is no established treatment indication for asymptomatic P-AVMs. In such cases, early treatment is undertaken based on concerns regarding future enlargement of the P-AVM, progression of chronic pancreatitis, and portal hypertension. We also determined that percutaneous embolization was minimally invasive and tolerable.

Given the nidus and small feeding arteries regressed over time rather than immediately post-embolization, only a single follow-up angiography was performed, after which surveillance was conducted with non-contrast CT, accepting coil-related artifacts. In both cases, during the 3-year postoperative period, no evidence of significant vascular recurrence was observed.

## Conclusion

In conclusion, transhepatic embolization for Yakes type IIIa or IIIb P-AVMs can be an effective approach. Understanding the detailed anatomy of each P-AVM is crucial for accomplishing this treatment. However, a limitation of this study was the short post-treatment follow-up period.

## Ethical declaration

For this type of study formal consent is not required.

## Date availability

Due to patient confidentiality and privacy regulations, the data supporting this study cannot be shared.

## Ethics declaration

Not applicable.

## Patient consent

Informed consent was obtained from all individual participants included in the study.

## Appendix Supplementary materials

Image, application 1
