# Supplementary material for: Two cases of pancreatic arteriovenous malformations treated with coil embolization via percutaneous transhepatic portal vein approach
Source: Radiol Case Rep. 2025 Oct 8;21(1):138–43. doi: 10.1016/j.radcr.2025.09.038 (PMC12546769; doi:10.1016/j.radcr.2025.09.038)
Supplement: Supplementary file 1 [file mmc1.pdf]

License Details

This Agreement between Yu Sasaki/ Department Radiology, University of Yamanashi Hospital ("You") and Elsevier ("Elsevier") consists of your license details and the terms and conditions provided by Elsevier and Copyright Clearance Center.

Print Copy

License Number	6087381102192
License date	Aug 13, 2025
Licensed Content Publisher	Elsevier
Licensed Content Publication	Techniques in Vascular and Interventional Radiology
Licensed Content Title	Interventional Management of Arteriovenous Malformations
Licensed Content Author	Gilles Soulez,MD Gilbert,MD Giroux,MD Racicot,Josée Dubois
Licensed Content Date	Dec 1, 2019
Licensed Content Volume	22
Licensed Content Issue	4
Licensed Content Pages	1
Type of Use	reuse in a journal/magazine
Requestor type	academic/educational institute
Portion	figures/tables/illustrations
Number of figures/tables/illustrations	1
Format	electronic
Are you the author of this Elsevier article?	Yes
Will you be translating?	Yes, including English rights
Number of translations	1
Title of new article	Two cases of pancreatic arteriovenous malformations treated with coil embolization via percutaneous transhepatic portal vein approach: A Case Report
Lead author	Yu Sasaki
Title of targeted journal	Radiology case report
Publisher	Elsevier
Expected publication date	Oct 2025
Portions	Table2
The Requesting Person / Organization to Appear on the License	Yu Sasaki/ Department Radiology, University of Yamanashi Hospital
Specific Languages	English
Requestor Location	3010-1<br>Shimokato<br>Nakakomagun<br>Chuo-shi, Yamanashi-ken 4093821<br>Japan
Publisher Tax ID	JP00022
Customer Tax ID	JP590475891939
Total	**0 JPY**

Copyright © 2025 Copyright Clearance Center, Inc. All Rights Reserved. Privacy statement. Data Security and Privacy. For California Residents. Terms and Conditions.
Comments? We would like to hear from you. E-mail us at customercare@copyright.com
